# LatAtk: A Medical Image Attack Method Focused on Lesion Areas with High Transferability

**DOI:** 10.3390/jimaging11110404

**Published:** 2025-11-11

**Authors:** Long Li, Yibo Huang, Chong Li, Fei Zhou, Jingjing Li, Kamarul Hawari Ghazali

**Affiliations:** 1 Joint International Research Laboratory of Spatio-Temporal Information and Intelligent Location Services, Guilin University of Electronic Technology, Guilin 541004, China; lilong@guet.edu.cn (L.L.); h19982410929@163.com (Y.H.); 2 Guangxi Key Laboratory of Digital Infrastructure, Guangxi Zhuang Autonomous Region Information Center, Nanning 530201, China; syszhoufei@163.com; 3 Key Laboratory of Equipment Data Security and Guarantee Technology, Ministry of Education, Guilin University of Electronic Technology, Guilin 541004, China; welcomerain@126.com; 4 School of Cyber Security, Jinan University, Guangzhou 510632, China; 5 Faculty of Electrical and Electronics Engineering Technology, Universiti Malaysia Pahang Al-Sultan Abdullah, Pekan 26600, Malaysia; kamarul@umpsa.edu.my

**Keywords:** adversarial machine learning, class activation mapping, deep learning, image texture, smart healthcare

## Abstract

The rise in trusted machine learning has prompted concerns about the security, reliability and controllability of deep learning, especially when it is applied to sensitive areas involving life and health safety. To thoroughly analyze potential attacks and promote innovation in security technologies for DNNs, this paper conducts research on adversarial attacks against medical images and proposes a medical image attack method that focuses on lesion areas and has good transferability, named LatAtk. First, based on the image segmentation algorithm, LatAtk divides the target image into an attackable area (lesion area) and a non-attackable area and injects perturbations into the attackable area to disrupt the attention of the DNNs. Second, a class activation loss function based on gradient-weighted class activation mapping is proposed. By obtaining the importance of features in images, the features that play a positive role in model decision-making are further disturbed, making LatAtk highly transferable. Third, a texture feature loss function based on local binary patterns is proposed as a constraint to reduce the damage to non-semantic features, effectively preserving texture features of target images and improving the concealment of adversarial samples. Experimental results show that LatAtk has superior aggressiveness, transferability and concealment compared to advanced baselines.

## 1. Introduction

Deep neural networks (DNNs) have been widely applied in fields such as lesion identification [1], disease diagnosis [2,3], and brain–computer interface [4] in smart healthcare, and have achieved a series of outstanding performances.

However, DNNs are susceptible to elaborate adversarial samples, i.e., by adding perturbations to input samples to make models give erroneous results with high confidence [5,6]. Additionally, adversarial samples can reveal sensitive information about the internal structure of models, which attackers can exploit for potential criminal activities. These issues have raised security concerns of deep learning applications in critical domains, especially in the medical field which is closely related to human health and well-being [7].

Adversarial attacks against image processing models are mainly aimed at traditional applications such as face recognition, but in recent years, some attack methods on natural images have also migrated to medical image processing. Ma et al. [8] employed four conventional adversarial attack methods to attack DNNs used for medical classification and found that even state-of-the-art medical classifiers are extremely vulnerable. Although their attack effect is remarkable, it should be noted that these attack methods are not specifically developed for medical images, leaving room for improvement in terms of attack concealment and success rate. First, most attack methods typically construct adversarial samples based on entire images rather than lesion areas in medical images, leading to extensive interference and reducing concealment of adversarial samples. Second, medical images often exhibit distinct texture features, but existing attacks have not fully utilized and adequately protected these features. Third, most existing methods assume that the attacked models are known (i.e., white-box attack), resulting in poor attack performance against unknown models (i.e., black-box attack). In other words, the transferability of such attacks is poor due to the suboptimal interpretability of DNNs [9]. However, Li et al. [10] demonstrated that features learned by intermediate layers of DNNs are transferable. Fourth, most attack methods target binary classification models, while medical images are diverse and often require multi-classification based on disease severity, diseased area, etc. Rosenblatt et al. [11] asserted that by applying tiny perturbations to biomedical data, model performance can be deceptively improved significantly. This “false performance improvement” attack is more covert and poses a greater threat than traditional performance-degrading attacks.

To address the aforementioned issues, a novel attack method focused on lesion areas with high transferability is proposed, named LatAtk. By utilizing class activation mapping (CAM) and texture features of medical images, LatAtk enhances the transferability and concealment of adversarial attacks in medical scenarios. Besides, it provides valuable insights for improving the security and robustness of DNNs for medical image processing.

Specifically, the contributions of this paper are as follows:

- LatAtk is proposed. First, a segmentation module is introduced to identify lesion areas in medical images that have a significant impact on classification results. Second, attack perturbations are only added to lesion areas and deep features of the classification model are fully utilized, constructing adversarial samples with less perturbations, and achieving efficient attacks on classification models.
- A loss function that considers both class activation loss and texture feature loss is proposed. The class activation loss enables LatAtk to disrupt the attention of surrogate models on images in classification space, enhancing the attack capability of adversarial samples on unknown models, i.e., possessing good transferability. The texture feature loss reduces the destruction of non-semantic features in images, enhancing the concealment of adversarial samples.
- Based on HAM10000 [12] and APTOS 2019 BD [13], comprehensive experimental evaluation and comparative analysis are conducted. Experimental results indicate that LatAtk demonstrates ideal attack effectiveness and transferability. In addition, ablation experiments demonstrate that image segmentation, class activation loss function and texture feature loss function in LatAtk are effective.

## 2. Related Work

This section aims to provide a concise yet comprehensive review of foundational research on adversarial attacks targeting natural images, along with their adaptations and extensions within the medical imaging domain.

### 2.1. White-Box Attacks

White-box attacks represent a prevalent assumption in adversarial attack research, wherein attackers possess complete knowledge of the target model, encompassing the training set, architecture, model type, and parameters.

Goodfellow et al. [6] pioneered the Fast Gradient Sign Method (FGSM), a gradient-based attack that rapidly increases classification loss by adding perturbations in the opposite direction of the gradient. Despite its speed in generating adversarial samples, FGSM’s calculated perturbations lack precision, resulting in a relatively low attack success rate (ASR). Kurakin et al. [14] refined FGSM by introducing multi-step perturbations and clipping adversarial samples to an effective region, ensuring they remain within an ϵ-neighborhood of the original image, thereby proposing an iterative FGSM (I-FGSM). Dong et al. [15] further enhanced ASR by integrating momentum terms into the iterative process, resulting in the Momentum-based I-FGSM (MI-FGSM), which stabilizes update directions and escapes poor local maxima, yielding more transferable adversarial samples. Wang et al. [16] proposed variance tuning MI-FGSM (VMI-FGSM). At each iteration for the gradient calculation, instead of directly using the current gradient for the momentum accumulation, VMI-FGSM further considers the gradient variance of the previous iteration to tune the current gradient so as to stabilize the update direction and escape from poor local optima. Although I-FGSM, MI-FGSM and VMI-FGSM outperform FGSM in terms of perturbation precision and ASR, they come at the cost of increased computational complexity.

In contrast to gradient-based attacks, Szegedy et al. [5] framed adversarial sample generation as an optimization problem, achieving attacks through solution. Madry et al. [17] explored adversarial robustness through robust optimization, proposing projected gradient descent (PGD) as a universal first-order adversary. Carlini et al. [18] introduced the CW (Carlini & Wagner) attack, defining and experimentally selecting optimal objective functions. Baluja et al. [19] proposed adversarial transformation networks, which generate adversarial samples by optimizing a joint objective function, ensuring similarity to source images while causing high-confidence misclassification by the target model.

### 2.2. Black-Box Attacks

Black-box attacks, another key assumption in adversarial attack, limit attackers to observing only the outputs of the target model for given inputs, without access to internal details. This more realistic scenario has become a focal point in adversarial attack research.

Xiao et al. [20] introduced AdvGAN, leveraging generative adversarial networks (GANs) to generate adversarial samples. Jandial et al. [21] enhanced AdvGAN by proposing AdvGAN++, which extracts convolutional layer features from the target model to fully utilize the potential features of original samples in adversarial sample generation. Wang et al. [22] proposed AT-GAN (Adversarial Transfer on GAN), a generative attack model that learns the distribution of original samples to generate adversarial samples with random perturbations, unrestricted by benign samples. Jiang et al. [23] introduced CycleAdvGAN, which trains two generators to generate and recover adversarial samples, respectively, integrating attack and defense in model training.

The transferability of adversarial samples, however, can be compromised by differences in architecture and training sets between the surrogate and target models (i.e., surrogate bias), weakening attack effectiveness. To mitigate this, Feng et al. [24] proposed a robust adversarial transferability mechanism against surrogate bias, transferring partial parameters of the conditional adversarial distribution from the surrogate model while learning untransferred parameters based on target model queries. Inkawhich et al. [25] demonstrated that adversarial samples with stronger transferability can be constructed in the feature space, generating adversarial samples by minimizing activation values at a specific model layer. Wang et al. [26] proposed a feature importance-aware attack, utilizing aggregated gradients to identify and disrupt critical features dominating the model’s decision-making process. Kim et al. [27] introduced an attention space-based attack, interfering with the model’s attention on images to disrupt critical decision-making features while exploring the entire search space to avoid local optima.

### 2.3. Adversarial Attacks in Medical Imaging

Despite significant advancements in adversarial attacks on natural images, research on their application to medical images remains limited, leading to an insufficient understanding of the unique threats they pose.

Existing studies on medical image adversarial attacks primarily focus on analyzing the robustness of DNN models in medical image processing and their ability to detect adversarial samples [3,8,28]. Many of these methods directly apply attack techniques developed for natural images to medical images, overlooking the distinct characteristics of medical images, such as variations in size, texture, and significant visual differences within the same disease category. This oversight results in less targeted and effective attacks.

Ma et al. [8] demonstrated that medical images are more susceptible to adversarial attacks than natural images, requiring fewer perturbations for successful attacks. They also noted the high detectability of adversarial samples in medical images, attributing it to extensive perturbations in areas outside lesion regions. To address these challenges, Qi et al. [29] proposed Stabilized Medical Image Attacks (SMIA), incorporating a loss deviation term to increase the difference between predicted and true values of adversarial samples and a loss stabilization term to ensure similar model predictions. Wang et al. [30] introduced an attack method utilizing classification space-based GANs to generate perturbations, focusing them on lesion areas through an attention mechanism to enhance adversarial sample concealment. For adversarial attacks on medical image segmentation, Cui et al. [31] showed that segmentation results can be manipulated by altering a small number of pixels, proposing DEAttack, which uses multiple iterative attacks based on differential evolution to automatically identify the most sensitive areas in input images. Unlike gradient-based methods, DEAttack maintains solution space diversity throughout the optimization process, preventing the algorithm from getting stuck in local optima.

The above attack methods can lead to unreliable predictions and poorly calibrated confidence, hence hindering clinical applicability. Therefore, in addition to attack techniques, research on model security and robustness is also increasingly emerging. Dong et al. [32] conducted the first systematic exploration of the robustness of deep learning models for medical diagnosis. They comprehensively reviewed the latest progress in adversarial attacks and defenses for medical image analysis based on application scenarios, and completed qualitative and quantitative analysis. In addition, they established a new and fair benchmark for evaluating the robustness of medical diagnosis models. Javed et al. [33] conducted an in-depth analysis of the factors (complexity, data, hyperparameters) and security threats (adversarial, privacy attacks) that affect the reliability of medical models, discussed defense measures such as adversarial training and preprocessing, and sorted out the tools and evaluation indicators for evaluating the reliability of technology. Shen et al. [34] proposed LaDiNE, the first solution that can effectively achieve both confidence calibration and adversarial robustness simultaneously. Specifically, transformer encoder blocks are used as hierarchical feature extractors that learn invariant features from images, resulting in features that are robust to input perturbations. In addition, diffusion models are used as flexible density estimators to estimate member densities conditioned on the invariant features, leading to improved modeling of complex data distributions while retaining properly calibrated confidence.

## 3. Proposed Method

This paper attempts to generate adversarial samples by combining image features with the interpretability of DNNs, so as to improve the attack capability of adversarial samples, especially against unknown models (i.e., to enhance the transferability of adversarial samples). To this end, a lesion area-constrained attack is proposed based on CAM and local binary patterns (LBP), which will be described in detail in this section.

### 3.1. Problem Description

Assuming that the classification model is represented as F(x)=y, where *x* represents the target image and *y* represents its true label. The goal of this study is to generate an adversarial sample $x^{adv}$ that leads to model misclassification (i.e., $F\left(x^{adv}\right)\neq y$) by adding a carefully crafted adversarial perturbation η to *x*, while still satisfying $H\left(x^{adv}\right)\neq y$ on the unknown target model *H*. To better balance the ASR of adversarial attacks and the maximum value of perturbation added while achieving the above goal, this paper formulates the generation of adversarial samples as an optimization problem, as shown below:

(1) $$\begin{array}{cc} \; & \underset{x^{\mathrm{adv}}}{\mathrm{argmax}}\;L\left(x^{\mathrm{adv}},y\right)\\ \; & \mathrm{s.t.}\;F(x)=y\\ \; & \;\;\;\;\;\;\eta =\mathrm{seg}(G_{\theta }\left(x\right))\\ \; & \;\;\;\;\;\;x^{\mathrm{adv}}=x⊕\eta \\ \; & \;\;\;\;\;\;F(x^{\mathrm{adv}})\neq y\\ \; & \;\;\;\;\;\;{∥\eta ∥}_{p}<\epsilon \end{array}$$

where seg() represents the segmentation operation, $G_{\theta }\left(x\right)$ represents the global adversarial perturbation generated by the generator *G*, θ is a key parameter of *G*, η is the adversarial perturbation after segmentation (or local adversarial perturbation), the operator ⊕ represents pixel-wise addition of images, and *p*-norm ${∥∥}_{p}$ and ϵ are used to measure and limit the amount of the perturbation, respectively.

### 3.2. Framework and Workflow of LatAtk

**Figure 1:**
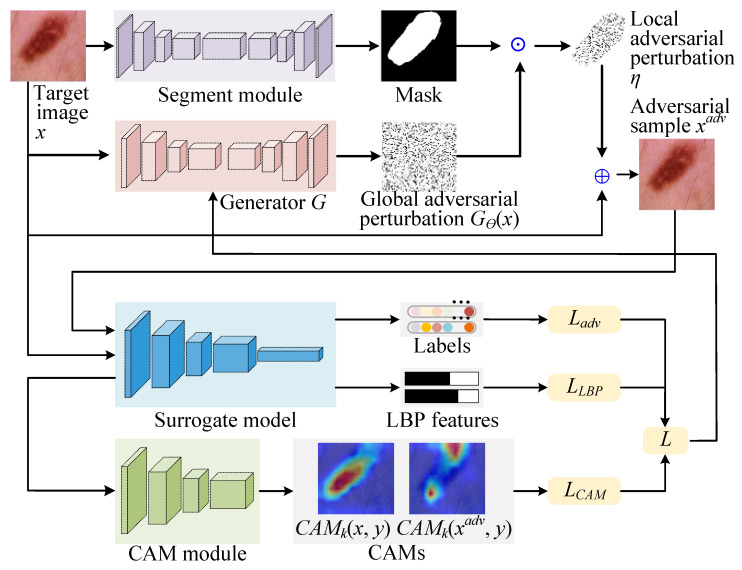
Workflow of LatAtk.

The framework and workflow of LatAtk are shown in Figure 1. Given a target image *x*, LatAtk will perform the following steps in sequence: (1) Identify the attackable area in *x* by segmenting the lesion area; (2) Initialize the adversarial perturbation and continuously optimize it based on the constraint function and interactions with the surrogate model; (3) Generate the final adversarial sample $x^{adv}$ for attacking classification networks.

#### 3.2.1. Attackable Area Identification Based on Lesion Area Segmentation

LatAtk uses U-Net to identify the lesion area in *x* and outputs two binary masks to identify the attackable area *T* while protecting the non-attackable region $\bar{T}$.

Specifically, given a medical image *x* of size w×h, $T=x⊙M_{k}$, and $\bar{T}=x⊙{\bar{M}}_{k}$. The operator “⊙” denotes Hadamard product (an element-by-element multiplication, also known as the element-wise product, entry-wise product or Schur product). $M_{k}$ and ${\bar{M}}_{k}$ are binary masks for attackable and non-attackable areas, with a size of w×h and elements with values of 0 or 1.

LatAtk uses the standard U-Net as the image segmentation module. It features a symmetric encoder-decoder structure, four downsampling and four upsampling stages, ReLU activations in each layer, and skip connections at each resolution. LatAtk trained this module from scratch on ISIC 2018 Task 1 (lesion masks), and evaluated the mask quality on the validation set, with the result that mean Dice ≈0.90 (IoU ≈0.83).

#### 3.2.2. Adversarial Perturbation Optimization Based on CAM and LBP

During the adversarial sample generation process, the generator *G* in LatAtk first initializes the adversarial perturbation randomly, and then optimizes the perturbation (including $G_{\theta }\left(x\right)$ and η) in collaboration with the surrogate model under given constraints. The optimized adversarial samples have the characteristics of strong transferability and imperceptible perturbation while achieving effective attacks.

In Figure 1, the CAM module is used to obtain class activation maps (CAMs) from the surrogate model during the adversarial sample generation process. LatAtk improves its transferability by maximizing the class activation loss between adversarial and target samples. The generator *G* minimizes the impact of adversarial perturbations on original samples by obtaining the output labels of target and adversarial samples on the surrogate model, as well as the LBPs that describes texture features, ensuring the imperceptibility of perturbations.

**Figure 2:**
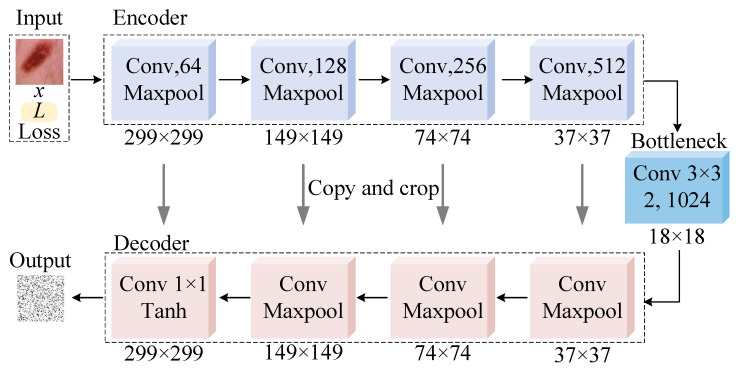
Architecture of Generator G.

In addition, the generator *G* employs an encoder-decoder architecture, as shown in Figure 2. The encoder employs 3 × 3 convolutional kernels and ReLU activation functions for feature extraction, coupled with 2 × 2 max pooling layers to progressively downsample the input and capture high-level semantic information. The decoder employs 2 × 2 transposed convolution for upsampling, along with 3 × 3 convolution kernels for feature refinement. The skip-connection mechanism is the core of this architecture, which concatenates the feature maps of each encoder layer with the corresponding decoder layer. This design effectively integrates low-level spatial details with high-level semantics, enabling *G* to produce perturbations that closely align with the structural characteristics of the input image *x*. The final output layer maps the number of channels through a 1 × 1 convolution, and strictly constrains the perturbation amplitude within the range of [−1, 1] using the Tanh() activation function. *G* demonstrates excellent performance of LatAtk in black-box attack scenarios through end-to-end training.

#### 3.2.3. Construction of Adversarial Samples

To reduce changes to *x* and increase the concealment of the attack, LatAtk only attacks the area corresponding to the binary mask $M_{k}$, rather than the entire image. Specifically, LatAtk adds the adversarial perturbation η generated by *G* to the attackable area *T* of *x*, thereby constructing the adversarial example $x^{adv}$, represented as:

(2) $$x^{adv}=x⊙{\bar{M}}_{k}⊕\left(x⊕G_{\theta }\left(x\right)\right)⊙M_{k}$$

### 3.3. Acquisition and Utilization of CAMs

To enhance the aggressiveness and transferability of adversarial attacks, LatAtk focuses on areas that are more critical for model classification and features that play a decisive role in model decision-making. Gradient-weighted CAM (Grad-CAM) [35] is used to visualize and understand the decision-making process of DNNs. Its main idea is to obtain the importance of each feature map in influencing model decision-making by calculating the gradient of the target category score relative to the feature map of the last convolutional layer.

For a medical image *x* of size w×h, the feature importance $CAM_{k}$ is defined as:

(3) $$CAM_{k}\left(x,y\right)=\mathrm{ReLU}\left(a_{k}^{c}\cdot A^{k}\right)$$

(4) $$a_{k}^{c}=\frac{1}{hw}\sum_{i=1}^{h}\sum_{j=1}^{w}\frac{\partial l^{y}}{\partial A_{ij}^{k}}$$

where $l^{y}$ represents the logit output corresponding to the true label *y*, $A^{k}$ represents the activation value of the feature map at layer *k*, and $a_{k}^{c}$ represents the neuron importance weight obtained by performing global average pooling on gradients returned by the model.

From the above formula, it can be concluded that the importance of a feature is proportional to its impact on model decision-making. Therefore, the generator *G* in LatAtk disrupts deep features that have a positive effect on decision making based on their importance, which is achieved by maximizing the following loss function during training:

(5) $$L_{CAM}={∥CAM_{k}\left(x,y\right)-CAM_{k}\left(x^{adv},y\right)∥}_{2}$$

### 3.4. Preservation of Texture Features

In adversarial attacks, perturbations may destroy images on a large scale, resulting in easily noticeable changes. To minimize the differences between adversarial samples and target images, LatAtk utilizes LBP to maintain their visual consistency. LBP is an effective texture feature description method with advantages such as rotation invariance and grayscale invariance. LBP operator is usually defined in a window of equal length and width, and the texture features of the entire image is obtained by comparing the grayscale values of the central pixel with its adjacent pixels.

(6) $$LBP\left(c\right)=\sum_{q\in Q}s\left(I\left(q\right)-I\left(c\right)\right)2^{q}$$

(7) $$s\left(a\right)=\left\{\begin{array}{cc} 1, & a\geq 0\\ 0, & \mathrm{otherwise} \end{array}\right.$$

In the above equations, *c* represents the center pixel of the image window, *q* represents the *q*-th adjacent pixel of *c*, *Q* represents all adjacent pixels of *c*, I() represents the grayscale value of a certain pixel, and s() is the sign function.

The LBP operator in LatAtk is defined within a 3 × 3 window. By using the grayscale value of the central pixel in the window as the threshold and comparing it with the grayscale values of the 8 adjacent pixels, an 8-bit binary number can be obtained. The decimal LBP code obtained by converting this binary number can reflect the texture features of the image.

During the training process, LatAtk obtains the grayscale images of the target image *x* and the adversarial sample $x^{adv}$, which are used to generate LBP(x) and $LBP\left(x^{adv}\right)$. The texture features of *x* are preserved as much as possible by minimizing the following constraint function:

(8) $$L_{LBP}={∥LBP\left(x^{adv}\right)-LBP\left(x\right)∥}_{2}$$

### 3.5. Loss Function

To achieve more effective adversarial attacks, the loss function in LatAtk is defined as follows:

(9) $$L=-L_{adv}-\lambda_{CAM}L_{CAM}+\lambda_{LBP}L_{LBP}$$

where $\lambda_{CAM}$ and $\lambda_{LBP}$ are weighting coefficients, and J() is the cross-entropy loss.

(10) $$L_{adv}=J\left(F\left(x^{adv}\right),y\right)$$

Intuitively, the loss *L* consists of three parts: adversarial classification loss $L_{adv}$, class activation loss $L_{CAM}$, and texture feature loss $L_{LBP}$. In addition, it should be explained that Equation (1) theoretically expresses the maximization objective of LatAtk, while Equation (9) minimizes its inverse (i.e., loss) based on the standard gradient descent algorithm.

The specific values of $\lambda_{CAM}$ and $\lambda_{LBP}$ are determined by trial and error. The detailed procedure is as follows: First, based on a preliminary understanding of the task characteristics and an estimation of the role of CAM and LBP features in LatAtk, we set initial weight values. We preliminarily believe that CAM features are more critical for classification tasks, so we assign them a relatively large initial weight of $\lambda_{CAM}$ = 0.7. LBP features mainly provide local texture information and have a relatively small auxiliary effect on overall classification, so we set $\lambda_{LBP}$ = 0.3. Second, we conducted a series of experiments on a validation set. Each experiment only changes one weight value, keeping the other weight unchanged, and observes LatAtk’s performance on the validation set. Through multiple such univariate experiments, we gradually adjusted the weights and observed the changing trend of performance indicators. When further adjustments to a certain weight no longer improved performance, or even caused performance decline, we considered that a relatively suitable weight combination is found. After multiple rounds of adjustments, the model achieved ideal results when $\lambda_{CAM}$ = 1 and $\lambda_{LBP}$ = 0.5.

Different from previous methods that disrupt attention heatmaps [27,36], LatAtk applies per-channel normalization after adding adversarial perturbations to *x*. This is mainly due to the following considerations: (1) Feature independence. In LatAtk, the features from different channels capture different aspects of the image, i.e., some channels may focus on color information, while others focus on texture or edge information. Per-channel normalization ensures that the features from each channel are adjusted within their own scale range, avoiding mutual interference between features from different channels, thus better preserving the independence of each channel. (2) Feature distribution. Feature distributions vary significantly across channels, but global normalization unifies these features across all channels within a global mean and variance range. This may disrupt the original feature distribution characteristics of certain channels, thereby hindering the model’s utilization of these features. In contrast, per-channel normalization can handle each channel separately, which is more in line with the actual characteristics of features and helps the model learn more accurate feature representations. Overall, per-channel normalization performs better than global normalization, and therefore is adopted in LatAtk.

The overall workflow of LatAtk is summarized in Algorithm 1. In this algorithm, Lines 1–2 initialize the generator $G_{\theta }$ and build the DataLoader on the dataset D with batch size *B* and random shuffling. Lines 3–5 iterate over epochs and mini-batches, where each mini-batch (x,y) is used to compute the attention mask *M* on F(x), identifying lesion-related attackable regions. Line 6 generates the perturbation $\eta =G_{\theta }\left(x\right)⊙M$, which restricts the adversarial modification to these regions. Line 7 constructs the adversarial image $x^{adv}={\mathrm{Clip}}_{x,\epsilon }\left(x+\eta \right)$, ensuring that the perturbation magnitude does not exceed the bound ϵ. Lines 8–11 compute the total training loss, including the adversarial classification loss $L_{adv}$, the attention constraint loss $L_{CAM}$, and the texture similarity loss $L_{LBP}$, weighted by coefficients $\lambda_{CAM}=1$ and $\lambda_{LBP}=0.5$. Line 12 updates the generator parameters via gradient descent with learning rate lr. Finally, after all iterations, the optimized generator $G_{\theta }$ produces adversarial samples $x^{adv}$ that are both effective and visually coherent.    
**Algorithm 1:** Constructing adversarial samples $x^{adv}$ using LatAtk
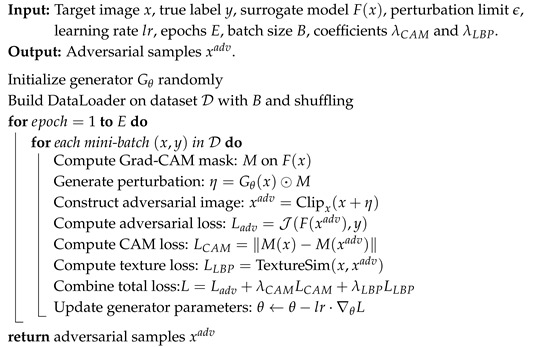


## 4. Results

### 4.1. Experimental Settings

All experiments in this paper are conducted on a GPU rental platform named AutoDL (https://www.autodl.com (accessed on 20 September 2025)). In terms of hardware, the selected computing platform includes an RTX 3090 24 GB GPU, an Intel(R) Xeon(R) Platinum 8350C 2.60 GHz CPU, and 42 GB of RAM. In terms of software, the operating system is Ubuntu 18.04, the deep learning environment is configured with PyTorch 1.8.1 and CUDA 11.1, and the programming language is Python 3.8.

### 4.2. Datasets and Neural Networks

#### 4.2.1. Datasets

The datasets selected in the experiment are HAM10000 [12] and APTOS 2019 BD (Asia Pacific Tele-Ophthalmology Society 2019 Blindness Detection) [13].

HAM10000 is a large collection of multi-sources dermatoscopic images of common pigmented skin lesions, which is released by the International Skin Imaging Cooperation (ISIC). Specifically, during the training of the segmentation module, HAM10000 2018 Task 1 is used, which contains 2594 training images, 1000 test images and their corresponding true masks. During the construction of adversarial samples, HAM10000 2018 Task 3 is used, which contains more than 10,000 images belonging to 7 categories of diseases: (1) actinic keratosis/Bowens disease (AKIEC), (2) basal cell carcinoma (BCC), (3) benign keratinous lesion (BKL), (4) dermatofibroma (DF), (5) melanoma (MEL), (6) melanocytic nevus (NV) and (7) vascular lesion (VASC). In addition, these diseases are further divided into “benign” and “malignant” tumors. Its detailed information of HAM10000 2018 Task 3 is shown in Table 1.

APTOS 2019 BD is an open (Kaggle) competition, the associated dataset contains 3662 retinal images. The dataset is organized by Aravind Eye Hospital, India, with the purpose of building an ML model to detect blindness autonomously without medical screening. These RGB images were captured using fundus photography over a long period of time under varying conditions and environments. Later, a group of trained doctors reviewed and labeled the gathered samples following the principle of the International Clinical Diabetic Retinopathy Disease Severity Scale (ICDRSS). As per the scaling system, the APTOS 2019 BD samples are divided into five categories: no Diabetic Retinopathy (DR), mild DR, moderate DR, severe DR, and proliferative DR, as shown in Table 2.

#### 4.2.2. Settings of Neural Networks

LatAtk is tested using image classification models pre-trained on ImageNet, including VGG16, ResNet50, DenseNet, and Inception_V3. In addition, a new fully connected layer is used to replace the original classifier to classify multiple categories of diseases.

Unless otherwise specified, all models in subsequent experiments were trained using the same hardware and software. The Adam optimizer is used to minimize the loss function during the training of neural networks, with a learning rate of $5\times 10^{-5}$, a weight decay coefficient of $1\times 10^{-5}$, and a batch size of 32. The generator was trained for 50 epochs, the adversarial perturbation amplitude ε was 0.01, and the input images were normalized to maintain numerical stability. These hyperparameter configurations demonstrated stable convergence and good generalizability across multiple experiments, providing a fair experimental basis for LatAtk and its comparative methods.

### 4.3. Baselines and Comparison

To demonstrate the effectiveness of LatAtk, it is comprehensively compared with state-of-the-art attack methods on medical images.

- FGSM [6]: A gradient-based adversarial attack method. It maximizes loss in the opposite direction of the gradient descent of the intermediate variable and adds a certain amount of perturbations to the image, achieving untargeted attacks.
- MI-FGSM [15]: During each iteration, momentum is introduced to balance directions of the current gradient and its previous gradient, ensuring that the generated adversarial samples are coherent in the perturbation direction.
- VMI-FGSM [16]: To further stabilize the gradient direction for reducing update oscillation, VMI-FGSM adds variance tuning into MI-FGSM.
- PGD [17]: Generates adversarial samples by iterating in the opposite direction of the gradient. To ensure the legitimacy of perturbations, the sample is projected after each iteration to ensure the perturbation remains within the allowed input space.
- CW [18]: An optimization-based attack method known for the imperceptibility of perturbations.
- SMIA [29]: An attack method designed to continuously generate medical adversarial samples, which consists of a loss deviation term and a loss stabilization term. The loss stabilization term smooths individual points by exhaustive searching the perturbation space, avoiding falling into local optima.

As for the complexity of these methods, it can be analyzed and compared theoretically. Let $F_{S}$ and $B_{S}$ represent the computational costs of forward propagation and backward propagation of surrogate models, respectively; $F_{G}$ represents the forward propagation computational cost of generator; *T* represents the number of iterations required for gradient based iterative attacks. For traditional iterative attack methods such as PGD, MI-FGSM, SMIA, VMI-FGSM, and CW, each iteration requires at least one forward and one backward propagation, resulting in an overall computational cost of $o(T\left(F_{S}+B_{S}\right))$. For single step attack methods such as FGSM, their complexity is $o(F_{S}+B_{S})$. LatAtk replaces the iterative optimization process with a trained generator that can generate perturbations through only one forward propagation. Therefore, in the inference stage, its time complexity is $o(T\left(F_{S}+B_{S}+F_{G}\right))$. Due to the fact that $F_{G}$≪$F_{S}$+$B_{S}$, LatAtk has a lower and fixed computational cost compared to any iterative attack with T>1.

### 4.4. Metrics

In terms of evaluation metrics, the performance of attack methods is measured from three perspectives.

#### 4.4.1. ASR

ASR, also known as fooling rate (FR), is commonly used to evaluate the strength of white-box attacks and the transferability of black-box attacks. Therefore, in this experiment, when the target model is consistent with the surrogate model, ASR is used to evaluate the strength of attacks; when the target model is different from the surrogate model, ASR is used to evaluate the transferability of attacks. In this paper, ASR refers to the proportion of adversarial samples that successfully deceive the model and make it output incorrectly, which can be calculated by the following formula.

(11) $$\mathrm{ASR}=\frac{\left|\{x^{adv}|F\left(x\right)=y\cap H\left(x^{adv}\right)\neq y\}\right|}{\left|\{x^{adv}\}\right|}\times 100\%$$

#### 4.4.2. CAMs

By visually displaying CAMs, the misleading effect of perturbations added by LatAtk on model output can be explained intuitively. Specifically, with the help of Grad-CAM, the features in the last convolutional layer of DNNs that have the greatest impact on classification are located in corresponding image areas, and their contribution to classification is mapped to original images in the form of heatmaps.

#### 4.4.3. Structural Similarity Index Measure (SSIM)

SSIM is a metric that measures the similarity between two images. In this study, SSIM is used to measure the similarity between target samples and adversarial samples, which directly reflects the concealment of adversarial attacks.

Given two images a and b, the SSIM can be calculated by the following formula:

(12) $$\mathrm{SSIM}\left(a,b\right)=\frac{\left(2\mu_{a}\mu_{b}+c_{1}\right)\left(2\sigma_{ab}+c_{2}\right)}{\left(\mu_{a}^{2}+\mu_{b}^{2}+c_{1}\right)\left(\sigma_{a}^{2}+\sigma_{b}^{2}+c_{2}\right)}$$

where $\mu_{a}$ and $\mu_{b}$ are average values of the data in *a* and *b*, $\sigma_{a}^{2}$ and $\sigma_{b}^{2}$ are variances, $\sigma_{ab}$ is the covariance $c_{1}$ and $c_{2}$ are constants used to maintain stability (to avoid denominators being 0). Note that −1≤SSIM(a,b)≤1, and SSIM(a,b)=1 if and only if a=b. More generally, the larger the SSIM, the more similar the two images are.

### 4.5. Comparison and Analysis of Experimental Results

#### 4.5.1. Comparison of ASRs

In the experiments, four typical convolutional neural network models (VGG16, ResNet50, DenseNet, and Inception_V3) pre-trained on the ImageNet dataset are selected for scheme evaluation. Several adversarial attack methods (FGSM, CW, PGD, MI-FGSM, VMI-FGSM, and SMIA), as well as the LatAtk proposed in this study, are used to generate adversarial samples. And then, the multi-classification and binary classification models constructed based on the above pre-trained models are attacked respectively. In addition, the Ensemble model (i.e., the average of the results from all target models) is constructed to validate schemes’ average attack capability in comprehensive scenarios.

In terms of parameter settings, based on research experience in the existing literature, 0.1 is selected as the maximum value of adversarial perturbations in the experiment, which is different from many attack methods targeting natural images. This is because medical images are easier to attack than natural images. In addition, the number of iterations of CW, PGD, MI-FGSM and SMIA are set to 50, 40, 10 and 10, respectively.

(1) Attacks on multi-classification models

For multi-classification tasks, the ASRs from attack experiments conducted on HAM10000 and APTOS 2019 BD are shown in Table 3 and Table 4.

Based on the results presented in Table 3 and Table 4, it can be observed that, in the multi-classification tasks conducted on the HAM10000 and APTOS 2019 BD datasets, different attack methods exhibit significant differences in attack success rate (ASR) and transferability. The ASRs of FGSM are generally the lowest (mostly below 25%), which is consistent with the limitations of its single-step gradient update mechanism. PGD and MI-FGSM achieve higher ASRs through iterative optimization and momentum mechanisms; however, their transferability is considerably restricted when there are large structural differences between the surrogate and target models (e.g., ResNet50→DenseNet or VGG16→Inception_V3). VMI-FGSM stabilizes the update direction through variance regularization, resulting in a slight improvement in ASR but theoretically increasing computational cost. The CW method minimizes perturbation perceptibility by optimizing perturbation magnitude, thereby generating visually more imperceptible adversarial examples, though its attack strength remains relatively weak. SMIA performs relatively stably across both datasets, yet its cross-architecture transfer performance is limited, with ASR reductions of approximately 4–6% on HAM10000 and 5–30% on APTOS 2019 BD. Compared with FGSM, CW, PGD, MI-FGSM, VMI-FGSM, and SMIA, LatAtk consistently maintains high and stable ASRs in most experiments, demonstrating superior attack strength and transferability. Specifically, across different combinations of surrogate and target models, LatAtk improves ASR by an average of 16.24–36.90% on HAM10000 and 4.54–39.48% on APTOS 2019 BD.

Furthermore, the results indicate that the choice of surrogate model plays an important role in transferability. Compared with other surrogate models, adversarial examples generated using Inception_V3 and VGG16 as surrogate models exhibit stronger cross-model transferability. For instance, on APTOS 2019 BD, when Inception_V3 is used as the surrogate model, its average ASR reaches 92.63%, exceeding other baselines by 6.21–48.22%. When VGG16 is used as the surrogate model, LatAtk achieves an average ASR of 91.14%, outperforming other baselines by 4.18–45.13%. In contrast, when DenseNet serves as the surrogate model, its average ASR is 86.25%, which is approximately 2% lower than that of PGD but still 3.06–31.77% higher than those of other baseline methods.

In addition, the characteristics of different datasets also influence the experimental results and help explain these performance variations. The HAM10000 dataset contains diverse lesion textures and relatively small lesion regions, making it difficult for baseline methods to focus on key discriminative areas, resulting in larger ASR fluctuations and limited transferability. In contrast, LatAtk effectively leverages lesion-area features guided by class activation maps (CAM) and texture constraints based on local binary patterns (LBP), leading to superior ASR and transferability performance. On the APTOS 2019 BD dataset, the image structures are relatively homogeneous, and the lesion regions are larger and more spatially distributed, which yields more stable performance across methods. Nevertheless, LatAtk still achieves outstanding results (average ASR = 90.30%), since these characteristics make lesion-region perturbations easier to generalize. This further verifies that the proposed method exhibits strong adaptability across different types of medical imaging data (e.g., dermoscopic and fundus color images).

(2) Attacks on binary classification models

Although LatAtk shows outstanding performance in transferability, its performance varies across different models. For example, ASRs of adversarial samples generated based on Resnet50 fluctuates between 45.67% and 85.96%. This is because ASRs are related to models’ classification ability, model structure, and feature distributions of data. Therefore, in addition to experiments on multi-classification models mentioned above, experiments on binary classification models are also conducted. Specifically, samples from HAM1000 and APTOS 2019 BD are reorganized according to “benign” and “malignant” labels of diseases, and the neural network models are modified to achieve binary classification tasks.

For binary classification tasks, the ASRs from attack experiments conducted on HAM10000 and APTOS 2019 BD are shown in Table 5 and Table 6.

Table 5 and Table 6 show that, in the binary classification tasks, the overall performance trends of different attack methods are largely consistent with those observed in the multi-classification experiments. FGSM achieves the lowest ASRs; PGD and MI-FGSM achieve moderate improvements, but their transferability remains limited; VMI-FGSM slightly improves ASR; CW and SMIA perform relatively stably, yet both exhibit weak transferability in cross-architecture attacks. LatAtk significantly outperforms all other methods across both datasets. Specifically, on HAM10000 and APTOS 2019 BD, LatAtk achieves average ASRs of 37.44% and 89.00%, respectively, surpassing other methods by 1.45–22.29% and 2.05–27.34%. These results demonstrate its superior attack capability and transferability. Regarding the effects of surrogate model selection and dataset characteristics, the results of the binary classification tasks are generally consistent with those observed in the multi-classification experiments and are therefore not elaborated here.

However, compared with the multi-classification tasks, the performance of LatAtk slightly declines in the binary classification tasks, mainly due to differences in the distribution of sample features. When multi-class samples are reclassified into two categories, inter-class differences are reduced while intra-class differences increase. This phenomenon leads to less distinct decision boundaries in binary classification models compared to multi-class ones, making it more difficult for adversarial samples to cross the boundary and thereby reducing attack effectiveness. Overall, the advantages of LatAtk in terms of ASR and transferability remain consistently robust and extend from multi-classification to binary classification tasks.

Based on the results in Table 3, Table 4, Table 5 and Table 6, the following conclusions can be drawn. (1) LatAtk achieves ideal ASRs and robustness. Whether in multi-classification or binary classification tasks, its ASRs is generally 20–40% higher than other methods, reflecting its high accuracy and strong transferability in adding perturbations to key features. (2) DenseNet and Inception_V3 are superior surrogate models. These two models have deeper feature hierarchies and denser connections, making the generated perturbations contain richer semantic information, which are more effective in transfer attacks. (3) The stable performance of LatAtk indicates its good generalizability. Whether it is dermoscopic images (HAM10000) or fundus color photos (APTOS 2019 BD), LatAtk can maintain high ASRs, indicating that it is not only suitable for specific tasks, but also has the potential to be promoted to a wider range of medical imaging fields. In summary, by combining local feature perturbations, global semantic interference, and sample adaptation strategies, LatAtk significantly improves attack efficiency while ensuring the perturbations are imperceptible. It shows consistent advantages across diverse datasets, models and tasks, verifying its effectiveness, robustness and versatility.

#### 4.5.2. Comparison of CAMs

**Figure 3:**
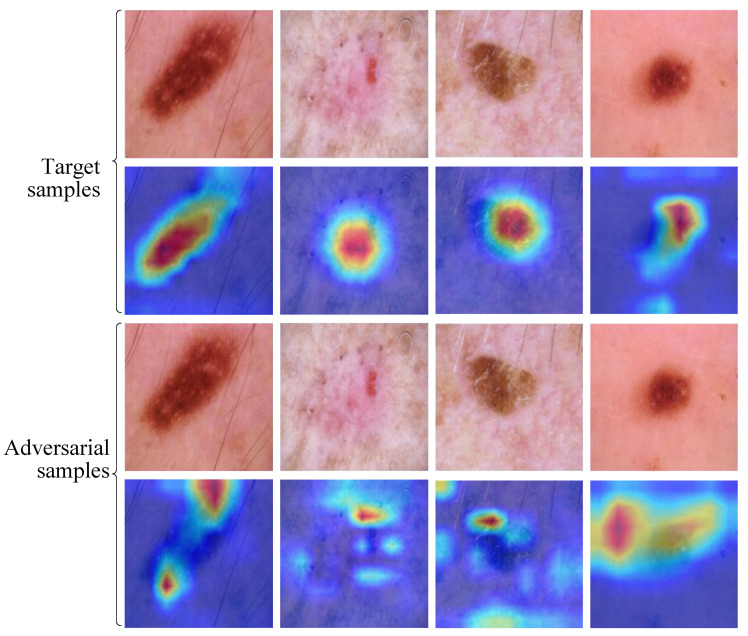
Samples and their corresponding CAMs.

To further explore the mechanism of DNNs for disease classification and the impact of small perturbations on model decision-making, this paper focuses on analyzing the changes and effects in class activation features of adversarial samples. The experiment is implemented by obtaining CAMs. The experimental results are shown in Figure 3, where the first two rows show target samples and their corresponding CAMs, and the last two rows show the adversarial samples and their corresponding CAMs. The redder the area in CAMs, the greater the role it plays in model decision-making.

The following conclusions can be drawn from the results in Figure 3: (1) When classifying medical samples, DNNs primarily focus on lesion areas (i.e., lesion areas dominate the model decision-making), which also verifies the rationality of LatAtk. (2) Compared with CAMs of target images, CAMs of adversarial sample changes significantly, indicating that adversarial perturbations added by LatAtk can effectively interfere with the attention of DNNs. (3) LatAtk’s attack on lesion areas will reduce their influence on model decision-making, thereby indirectly increasing the importance of non-lesion areas. In other words, LatAtk can induce models to focus on areas and features that are less relevant to correct classifications thereby reducing prediction performance. (4) Since different models will learn similar sample features during decision-making, LatAtk can successfully attack unknown classification models (i.e., it has ideal transferability), which is consistent with the experimental results shown in Table 3, Table 4, Table 5 and Table 6.

#### 4.5.3. Ablation Experiment

**Figure 4:**
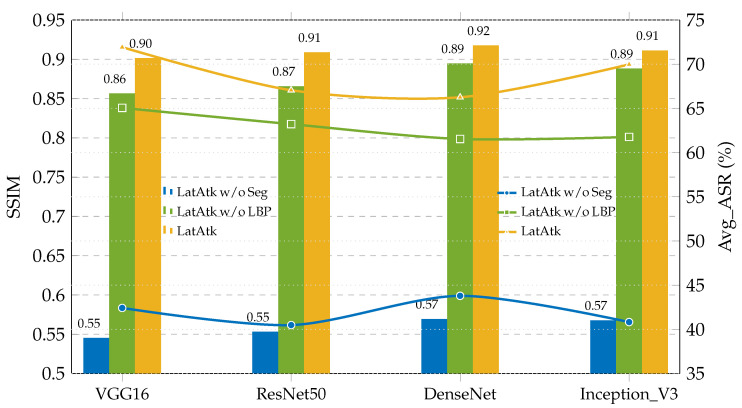
Results of ablation experiment.

This section will analyze the proposed LatAtk with the help of ablation experiments, especially revealing the impact of the segmentation module and the texture constraint ($L_{\mathrm{LBP}}$) on LatAtk. Specifically, four surrogate models (VGG16, ResNet50, DenseNet, and Inception_V3) are used to construct adversarial samples; The compared schemes include LatAtk, LatAtk without the segmentation module (LatAtk w/o Seg), and LatAtk without the texture constraint (LatAtk w/o LBP); SSIM and ASR are used to quantitatively measure and compare the performance of different schemes, where SSIM measures the visual consistency between adversarial samples and target samples, and ASR measures the attack effectiveness of schemes (i.e., the vulnerability of the model under adversarial perturbations). It should be noted that, since each surrogate model will attack multiple target models based on two datasets (HAM10000 and APTOS 2019 BD), to simplify the presentation of experimental results, we averaged the multiple ASRs corresponding to the same surrogate model to obtain a single but comprehensive metric, Avg_ASR, which is presented as the experimental result. The experimental results are shown in Figure 4.

In Figure 4, the bar chart represents SSIM and the line chart represents Avg_ASR. From the experimental results, it can be seen that: (1) Compared with LatAtk w/o Seg, LatAtk injects perturbations in smaller regions, thus constructing adversarial samples with greater visual concealment (corresponding to larger SSIMs). Due to the lack of regional constraints, the perturbations added by LatAtk w/o Seg cause greater damage to the images (corresponding to smaller SSIMs). (2) Compared with LatAtk w/o LBP, LatAtk constructs more covert adversarial samples (corresponding to larger SSIMs) by considering and preserving more texture features. Due to the lack of texture constraints, LatAtk w/o LBP cause greater damage to the images (corresponding to smaller SSIMs). (3) LatAtk has higher Avg_ASRs, indicating that targeted attacks on lesion areas enhance the effectiveness of attacks, and adding texture constraints helps construct more aggressive adversarial samples. In short, LatAtk performs best in SSIM and Avg_ASR on all surrogate models, indicating its strong attack capability while maintaining the visual consistency of images.

#### 4.5.4. Performance Comparison of Neural Network Models

In addition to the performance evaluation of the attack techniques described above, this study also analyzed the performance of different neural network models on the HAM10000 and APTOS 2019 BD datasets. The experimental results are shown in Table 7.

Evaluation metrics such as Accuracy, Precision, Recall, and F1 score are used to quantitatively display the experimental results. With the exception of Accuracy, which reflects the overall prediction performance of the model, all other metrics are calculated as both macro-average and weighted-average. The macro-average metric calculates the average for each category separately, providing a fair measure of the model’s overall performance across all categories and is suitable for situations with uneven class distribution. The weighted-average metric, on the other hand, weights the results based on the number of samples in each category and better reflects the model’s actual performance on key categories. Specifically, Macro Precision, Macro Recall and Macro F1 measure the balance of a model’s performance across all categories, while Weighted Precision, Weighted Recall and Weighted F1 reflect the model’s comprehensive classification capabilities across the entire data distribution. By comparing these metrics, the generalizability performance and stability of the model can be comprehensively evaluated.

Table 7 shows that on HAM10000, DenseNet and Inception_V3 perform well, with accuracies exceeding 0.90 and Macro F1 scores reaching 0.8536 and 0.8172, respectively. These metrics are relatively balanced, demonstrating good classification performance. ResNet50 and VGG16 performed slightly inferior.

On APTOS 2019 BD, the performance of each model is slightly lower than that of HAM10000. ResNet50 and VGG16 achieve high accuracies (87.16% and 86.34%, respectively), with minimal change in Macro F1 scores. DenseNet and Inception_V3 show a significant decrease in performance, with Macro F1 scores of only approximately 0.65, indicating insufficient generalizability or inadequate feature extraction on APTOS 2019 BD.

Overall, DenseNet has significant advantages in skin lesion classification (HAM10000), while ResNet50 and VGG16 are more robust in fundus image classification (APTOS 2019 BD). In other words, different models perform differently on different datasets, and model selection needs to be balanced based on specific datasets and application scenarios.

## 5. Conclusions

By exploring attack techniques targeting medical image classification models, this work provides insights into enhancing the security and reliability of deep learning applications in the medical field and hopes to promote the healthy development of smart healthcare. The proposed LatAtk divides the target image into attackable regions (lesion areas) and non-attackable regions and further affects the attention of deep learning models by injecting perturbations into lesion areas, thereby reducing their decision-making capability. At the same time, the newly designed loss function effectively maintains the visual features of the target image, enhancing the concealment of the attack. Overall, LatAtk achieves desirable attack effects at a relatively low cost.

In the future, the following research work will be further explored. (1) Addressing unseen modalities in medical scenarios: Currently, there is a lack of comprehensive understanding regarding how LatAtk performs when faced with unseen medical modalities. To bridge this gap, we plan to collect and utilize a wider range of medical datasets encompassing various rare or emerging modalities. For instance, we will explore datasets related to cutting-edge medical imaging techniques such as hyperpolarized MRI or advanced optical coherence tomography. By doing so, we aim to assess the generalizability and effectiveness of LatAtk across different and potentially unfamiliar medical data types. (2) True black-box attacks with limited samples: In true black-box cases, the attacker has no prior information about the model’s architecture, parameters, or training data, and is further constrained by limited samples. We intend to delve into semi-supervised or unsupervised learning approaches to overcome these challenges. For example, we will investigate how to leverage self-supervised learning techniques to extract meaningful features from the limited available samples, enabling the generation of effective adversarial examples in a true black-box setting. (3) Robust adversarial defenses in medical contexts: While adversarial attacks pose significant threats, developing robust defense mechanisms is equally crucial, especially in the medical field where data privacy and security are of utmost importance. We will explore adversarial training methods tailored to medical models, taking into account the unique characteristics of medical data and tasks. For example, we will investigate how to design adversarial training schemes that can effectively improve the model’s anti-attack ability without compromising its performance on critical medical diagnosis or treatment prediction tasks.

Furthermore, it should be noted that attacks on medical models may raise ethical concerns. Without sufficient discussion and analysis, relevant technical research and open-source releases are highly likely to be exploited maliciously, potentially endangering patient privacy and safety, and introducing social issues such as bias and discrimination. Therefore, conducting targeted AI ethics research is crucial.

## Figures and Tables

**Table 1:** Sample Labels and Number in HAM10000 2018 Task 3.

Dataset	Sample Labels and Number						
Partitioning	MEL	NV	BCC	AKIEC	BKL	DF	VASC
Training set	1113	6705	514	327	1099	115	142
Validation set	21	123	15	8	22	2	3
Test set	171	909	93	43	217	44	35

**Table 2:** Sample Labels and Number in APTOS 2019 BD.

Dataset	Sample Labels and Number				
Partitioning	No DR	Mild DR	Moderate DR	Severe DR	Proliferative DR
Training set	1434	300	808	154	234
Validation set	172	40	104	22	28
Test set	199	30	87	17	33

**Table 3:** Attack Effects on Multi-classification Models (HAM10000).

Surrogate	Target	ASRs of Different Attack Methods (%)						
Models	Models	FGSM	CW	PGD	MI-FGSM	VMI-FGSM	SMIA	LatAtk
VGG16	ResNet50	18.23	24.53	22.92	31.25	57.81	18.75	77.08
	DenseNet	14.58	20.68	22.40	33.71	41.34	20.83	47.07
	Inception_V3	18.75	23.47	26.04	31.78	33.95	20.31	48.44
	Ensemble	13.54	20.68	22.92	31.78	38.14	17.71	45.01
ResNet50	VGG16	18.23	27.86	15.62	31.91	31.85	16.15	45.67
	DenseNet	18.23	25.66	23.96	33.98	41.34	18.23	85.96
	Inception_V3	16.15	24.00	15.62	34.18	33.95	13.02	52.60
	Ensemble	14.06	22.81	16.15	31.91	28.11	14.06	49.00
DenseNet	VGG16	19.27	26.06	18.23	31.25	31.85	19.27	48.60
	ResNet50	21.88	22.54	22.40	48.20	57.81	25.00	70.08
	Inception_V3	19.79	23.80	29.69	34.57	33.95	22.92	46.14
	Ensemble	16.67	20.74	22.40	32.45	29.71	19.27	48.00
Inception_V3	VGG16	17.71	27.86	20.83	31.85	26.66	21.35	57.25
	ResNet50	24.48	26.86	32.81	44.08	57.81	23.96	61.17
	DenseNet	20.83	24.27	31.25	37.50	41.34	21.88	42.75
	Ensemble	18.75	23.47	23.96	35.64	30.80	19.79	51.59

**Table 4:** Attack Effects on Multi-classification Models (APTOS 2019 BD).

Surrogate	Target	ASRs of Different Attack Methods (%)						
Models	Models	FGSM	CW	PGD	MI-FGSM	VMI-FGSM	SMIA	LatAtk
VGG16	ResNet50	71.04	70.49	93.44	74.86	40.27	82.79	89.95
	DenseNet	91.08	62.02	92.62	73.22	31.69	85.52	90.71
	Inception_V3	77.87	84.97	72.68	55.19	39.13	87.98	93.17
	Ensemble	80.05	54.37	89.07	76.23	72.95	87.70	90.71
ResNet50	VGG16	83.06	62.57	84.15	81.42	42.13	91.26	92.62
	DenseNet	70.22	65.30	57.92	66.94	39.40	70.77	87.43
	Inception_V3	93.17	90.44	89.07	89.62	64.92	77.60	90.44
	Ensemble	79.23	70.77	71.58	84.15	87.16	71.58	94.26
DenseNet	VGG16	52.19	78.14	75.68	53.01	36.28	94.54	78.42
	ResNet50	92.08	53.55	93.72	88.52	68.14	81.69	91.95
	Inception_V3	80.87	74.04	91.26	55.46	55.85	86.89	88.03
	Ensemble	89.34	66.12	92.35	81.69	57.65	69.67	86.61
Inception_V3	VGG16	75.14	73.50	92.35	77.05	47.65	63.66	91.26
	ResNet50	59.84	77.87	54.10	88.52	42.73	93.99	92.08
	DenseNet	91.26	92.62	65.85	74.32	41.31	93.99	93.44
	Ensemble	87.16	90.98	51.64	79.78	45.90	93.99	93.72

**Table 5:** Attack Effects on Binary Classification Models (HAM10000).

Surrogate	Target	ASRs of Different Attack Methods (%)						
Models	Models	FGSM	CW	PGD	MI-FGSM	VMI-FGSM	SMIA	LatAtk
VGG16	ResNet50	16.16	13.36	31.25	34.90	27.61	33.13	33.64
	DenseNet	18.55	13.36	39.06	33.33	32.70	39.58	36.44
	Inception_V3	20.81	15.62	35.42	34.90	27.01	37.50	39.06
	Ensemble	16.16	12.17	34.38	32.81	27.41	35.94	37.63
ResNet50	VGG16	18.28	17.15	30.73	33.33	26.66	32.29	34.31
	DenseNet	24.93	15.43	28.65	32.81	32.70	33.85	33.24
	Inception_V3	29.26	16.95	30.73	34.38	27.01	32.81	32.65
	Ensemble	19.75	13.03	30.21	31.77	25.61	31.25	31.25
DenseNet	VGG16	17.29	17.02	32.29	35.94	26.66	35.42	37.83
	ResNet50	27.66	16.09	36.98	40.10	27.61	42.71	44.75
	Inception_V3	24.34	15.96	36.98	35.42	27.01	36.98	35.84
	Ensemble	19.81	12.90	34.38	34.38	26.21	35.42	35.24
Inception_V3	VGG16	18.62	16.76	33.85	34.38	26.66	36.46	38.50
	ResNet50	28.19	16.29	37.50	36.98	27.61	38.54	50.20
	DenseNet	27.26	17.22	39.58	35.94	32.70	36.46	40.29
	Ensemble	21.41	13.10	33.33	33.85	26.21	35.42	38.10

**Table 6:** Attack Effects on Binary Classification Models (APTOS 2019 BD).

Surrogate	Target	ASRs of Different Attack Methods (%)						
Models	Models	FGSM	CW	PGD	MI-FGSM	VMI-FGSM	SMIA	LatAtk
VGG16	ResNet50	86.34	77.05	55.46	74.86	55.85	92.35	81.97
	DenseNet	86.07	52.73	72.40	90.98	69.95	74.04	87.87
	Inception_V3	89.07	85.52	93.99	91.53	75.19	93.72	94.48
	Ensemble	89.07	76.78	81.15	93.72	70.05	75.96	97.38
ResNet50	VGG16	89.62	60.93	61.20	81.69	69.67	82.51	83.66
	DenseNet	74.59	93.44	79.78	77.32	68.74	86.34	74.04
	Inception_V3	89.07	84.70	62.02	91.80	57.21	80.05	93.06
	Ensemble	69.13	93.72	64.48	85.25	71.31	87.98	93.66
DenseNet	VGG16	66.94	77.60	82.24	86.34	31.20	80.05	77.05
	ResNet50	91.80	77.87	78.69	65.57	45.19	91.80	91.97
	Inception_V3	87.16	87.16	81.69	57.92	58.58	73.22	88.31
	Ensemble	90.44	84.43	75.96	49.18	66.39	75.68	91.35
Inception_V3	VGG16	69.95	92.35	66.12	93.17	66.28	56.83	90.05
	ResNet50	92.35	87.98	89.89	89.07	63.77	72.95	91.15
	DenseNet	45.63	90.16	90.98	92.35	42.68	86.07	95.25
	Ensemble	84.43	91.53	91.80	92.90	74.48	90.16	92.79

**Table 7:** Performance of neural network models.

Datasets	Models	Accuracy	Macro	Macro	Macro	Weighted	Weighted	Weighted
			Precision	Recall	F1	Precision	Recall	F1
HAM10000	ResNet50	0.8892	0.8294	0.7954	0.8050	0.8876	0.8892	0.8853
	DenseNet	0.9111	0.8818	0.8306	0.8536	0.9116	0.9111	0.9108
	VGG16	0.8712	0.7831	0.7608	0.7683	0.8686	0.8712	0.8687
	Inception_V3	0.9011	0.8838	0.7803	0.8172	0.9006	0.9011	0.8993
APTOS 2019 BD	ResNet50	0.8716	0.7851	0.7526	0.7632	0.8727	0.8716	0.8697
	DenseNet	0.8169	0.7121	0.6285	0.6576	0.8063	0.8169	0.8051
	VGG16	0.8634	0.7894	0.7232	0.7455	0.8617	0.8634	0.8586
	Inception_V3	0.8197	0.7833	0.6286	0.6449	0.8295	0.8197	0.8037

## Data Availability

Data derived from a source in the public domain. The datasets used in this study are available in Github at https://github.com/johnyquest7/HAM10000 (accessed on 6 November 2025), and in Kaggle at https://www.kaggle.com/datasets/mariaherrerot/aptos2019 (accessed on 6 November 2025). Any additional data supporting the findings of this study are available from the corresponding author upon reasonable request.
